# On Mental Physiology
†Chapters on Mental Physiology. By Henry Holland, M.D., F.R.S. London: 1852.


**Published:** 1852-07-01

**Authors:** 


### Art. IV.—ON MENTAL PHYSIOLOGY.†

These chapters embrace the psychological portion of Dr. Holland's former able work, "Medical Notes and Reflections." We quote a passage from its preface, to indicate the value which the learned author places on the subject to which our journal is devoted:—"Scarcely can we name a morbid affection of the body in which some feeling or function of mind is not concurrently engaged. No physician can rightly fulfil his duties without an adequate knowledge of, and constant regard to, these important relations." It is refreshing to the mind to take up these pages of a philosopher in his unprejudiced pursuit of truth, after having been compelled to travel over, and, alas, to wade through, the pages of so many who have, in their course, blinded their eyes even to the facts around them, if opposed to their own notions; or who have precipitately formed crude deductions, and endeavoured to frame a gigantic theory on a foundation which is scarcely firm enough to support the most fragile hypothesis.

Convinced that it is our duty to proceed firmly in that course, which in the climax of its study may tend to render the term metaphysics a misnomer, we have ever, without any fear of the hackneyed stigma of materialism, regarded the investigation of the physio-pathology of the brain as the essence of psychological study, not by any shallow conclusions, *pro* or *con*, on popular prejudice or proselytism, but by a careful association of cerebral symptomatology with mental phenomena. That there are many things yet undreamed of in our philosophy must be admitted, and we must so far fail in our duty, even as the physicians of the body, when we blink or disregard psychal derangement, so often but a symptom of corporeal disease.

This must be done, however, without caprice or bigotry. We must accept every new and *proved* fact, and measure or explain it on the acknowledged principles of science; "separate," in Dr. Holland's own words, "what is known from that which is unknown—what is capable

† Chapters on Mental Physiology. By Henry Holland, M.D., F.R.S. London: 1852.

of being reached by the human understanding from that which is presumably unattainable by it." Thus restrained in our especial study, we are satisfied that we are progressing in the only path to the temple of truth.

With these sentiments, we at once recognise the importance of the first chapter, on medical evidence, especially in reference to the effect of remedy.

The idiosyncrasy of the mind, as well as that of the body, is as multiform as feature or shape; so, even the *truthful* evidence of one physician regarding his own favourite remedy, perhaps precipitately advanced from isolated cases, becomes a stumblingblock to the profession: *vide* the history of tar-water, digitalis, cubebs, iodine, &c. In the study of psychology this equally obtains, for insanity has its multiform phases; and even though the proximate cause or structural condition may be similar, its exciting causes are so various, and its sympathies so intricate, as to require equally patient study and discrimination regarding the principles of its treatment. Of the valuable hints and precepts of Dr. Holland, we may especially note those on the *jumble* and discrepancy of our medical nomenclature. In discussion, especially, a protracted war of words is often carried on upon one misapplied term; and when the strife is over, the subject is, of course, just as perplexing as ever, the combatants discovering that they have really been talking about two different things.

And this is aggravated by the prevailing fashions in medicine; as Dr. Holland says, "terms have descended to us which we can hardly put aside, maxims which fetter the understanding, and methods of classification which prevent the better suggestions of a sound experience."

Regarding our own pages, we have ever adopted the course here enjoined, of never rejecting what is new or strange, because it *is* new and strange. In proof of this we may refer to our criticisms on Reichenbach, and Mayo, and Crowe, and to our own original papers on psychal phenomena.

The faculty of attention, to which Dr. Holland refers, is one of the most important attributes of human intellect. The high degrees of its power constitute indeed the strong and energetic mind, and its physiology and pathology are often marked in the same individual. The concentration of the mind on one point, which has worked out some of the most abstruse problems, and the most marvellous works, may, by over-indulgence, become morbid abstraction or absence of mind, and the subject of very unmerciful satire: and if Sir Isaac Newton formed his brilliant theories from simple facts by "always thinking unto them," he was, it is said, from the same abstraction, guilty of very eccentric and even ridiculous behaviour. Indeed, *insanity* is not an infrequent consequence of the long continuance or excess of attention, which, like connate imbecility, cannot be fixed to a point. Consciousness is, indeed, for a time lost, as in the cases of Parmegiano and Archimedes, and the most strange illusions may also thus arise in the mind.

This principle of concentration is the grand secret of all the popular phenomena of the day, and it is almost the duty of the psychologist to develop this principle, so that the popular mind and purse may cease to be gulled and plucked. Whether this attention, or, as Dr. Holland would call it, direction of consciousness, or, as we have termed it, concentration of thought, be voluntary or reflex, as well as the nature of its intimate association with the phenomena of the day—all this is yet only on the threshold of development. It is this power of mental concentration on one point, which conferred on Archimedes, and Watt, and Smeaton, and Telford, and Stephenson, that wondrous power of working out and adapting mechanical forces to their will; and it is this concentration on another point which enables many to ward off an evil, or to endure torture with almost superhuman courage. It is the *in*voluntary or reflex influence of concentration, which excites the feats of fragile girls during the convulsive epidemics.

We have known, also, deep and fixed thought on one absorbing subject control or even obviate sea-sickness.

This concentration of the attention on one sense, will thus exalt and extend its power, but at the expense of the other senses. This is the rationale of community or transference of senses.

It is clear, from these reflections, of how much importance is the study of the influence of mind on body. As it *induces* disease by concentration, it may, when properly studied, prove of great remedial benefit, as in the cases of the Hohenlöhe *miracles*. Concentration of attention on the heart may instantly induce increased action, and, as in the case of Colonel Townsend, diminished action, even to a fatal extent. The principle will in the end, we think, be brought to bear as an important therapeutic agent.

Even concentrated thought on the intestinal canal will, as we have ourselves known, at once induce peristaltic action; and we are constantly aware how the contractions of the bladder obey the same influence. The thought, as well as the sight, of a savoury dish will directly excite the salivary glands to pour forth their fluid. The fixing of the thought will also induce disorder: the spasm of cramp may so be renewed after a contraction has ceased for a time.

There are some curious cases in which muscular action seems to depend on attention; for when it has been for an instant diverted, an article has immediately dropped from the hand. And this may even form one mode of the fulfilment of a prophecy.

Dr. Holland's reflections on that especial faculty so peculiar to man, the power of thinking of our thoughts, are interesting. We refer to "The influence of attention directed inwards upon those images or repetitions of objects of sense, which, even in the waking state, are perpetually generated within the sensorium, independently of all direct impressions from without, though often immediately consequent upon them."

Dr. Holland very candidly discusses the association of attention with the favourite phenomena of the day. We have, however, in the course of our labours, so fully discussed the subjects and effects of passes and odyle forces, the *virga divinatoria,* and other psychal novelties, that we can only refer our readers to the acute reasonings in our author's pages, in which the true balance is drawn between the material effects of magnetic force and the mere results of psychal influence—between the voluntary and automatic powers,—and to the many passages which, with the author's lucid phraseology, so constantly point out and explain the *cui bono* of what profane sceptics would call the imperfections of our nature; thus "vindicating the ways of God with man." The eccentricities which with the multitude constitute a complete puzzle, are thus clearly referred to: "In many remarkable cases the ordinary perceptions from the senses are wholly disturbed and perverted by the condition of the sensorium receiving them. Muscular motions occur from other causes than volition; and past images and memories rise up unbidden to perplex both sensations and acts by mingling with them, without control or direction of the rational will." Our own constant reflections (and Dr. Holland evidently coincides) convince us that the essence of mesmerism is in the body of the patient, and not in that of the operator: that is, the predisposing is of more account than the exciting cause, however essential the latter may be. The phenomena may, indeed, be brought out by many other modes than passes and touching, especially where there are weak or sensitive points. "A singular case is that of an expected impression on some part of the body producing, before actually made, sympathetic sensations or movement in other parts which are wont to be affected by such impressions." For the odyle force, Reichenbach chiefly selected females under 30, and all affected by some abnormal nervous condition. These females, we believe, are especially prone to deception, and there are few psychologists who will not at once recognise those to whom Dr. Holland refers, in Lord Bacon's words: "Delight in deceiving and aptness to be deceived, imposture and credulity, although they appear to be of a diverse nature, yet certainly they do for the most part concur."

The principle of consciousness—the feeling that *we are*—ah! who can answer the question of Dr. Holland? Who will ever fathom the mystery? Descartes, Berkeley, Locke, Hume, Priestley, Paley, all would look different ways to find it; and at last some vulgar Pyrrho might, with a "we wish you may get it," confute them all—not on the *truth* of personal existence, but because they could not find or prove it. Let us take Descartes. His *ego*—the *I-ness* or *ichikeit* of the Germans—is but a shallow definition of this principle. We are conscious we are, because we think—a notion, by the way, previously put by Milton into Adam's thought, "That I am I know, because I think." But the very *ego*, the thing that thinks, must have a prior existence; therefore, thinking is not the essence, although it may be the proof, of being. We quite agree with Dr. Holland that consciousness is a succession, and not simultaneousness. We believe that the slightest thought is an action: two, of course, cannot exist at the same time in the same part. It would be curious to *pit* this hypothesis of succession with one brain and the synchronous antagonism of the two brains of Wigan together; but we must, at least, defer it. There seems a constant antagonism between mental and physical consciousness, and intellect seems to hang on our power of making the former predominate. The idiot, when he is conscious, is only physically so; the perception exists, but it vanishes when the excitant is withdrawn.

This question of mental consciousness is of real consequence in psychology, as the method of arrangement of perceptions is at the root of all, constituting the varied degrees of intellect, from the idiot to the philosopher. So Cicero: "*Magni est ingenii revocare mentem a sensibus, et cogitationem a consuetudine revocare.*" And we might quote Samuel Johnson at Iona, were not his fine sentence as common as household words.

On this point of antagonism of will and senses Dr. Holland reasons with perspicuity, from whence we might, if we had space, found some very salutary precepts for the self-government of the mind, which might at once answer Dr. Holland's query.

Time Dr. Holland deems of much importance as an element in psychical studies, in regard to sanity, or health, or integrity of mind. We might contrast the closeness with which a question is answered, not only on recovery from adynamic or comatose states, but also in the early stages of typhus (the delirium of which so much resembles a dream), with those dreams in which a myriad of years is gone over in a few seconds—the one arising from inaptitude of perception or consciousness; the other from that exalted or concentrated condition of brain analogous to eccentric and almost preternatural muscular actions.

Dr. Holland's opinion coincides with that of Locke, that at one time the mind is in a *boggle*, and requires spurring; at others, it presses on like a war-horse, and cannot be held in. Walter Scott acted consciously on this principle of varied states and capacities of mind *quoad* time, when he was wont, after *trying* to recollect or compose in vain, to sleep on it, and it would all come in the morning; or when, to use his own graphic term, he had "*a fit of the clevers*." The misty brain, we may be sure, is known to all authors; even Milton was occasionally subject to it; while, at other times, "the unpremeditated verse flowed like an inspiration."

Probably not only the loss of memory in the senile brain, but also that of the incubation stage of insanity, may be thus explained: that is, ere the sluggish mind is conscious of a perception, or a question, so as to set about answering it, another idea or suggestion is forced on it, and thus, time being, as it were, called, the first idea is erased, and the answer does not come to the scratch. Some intellectual minds are thus set a wool-gathering. When John Kemble had indulged in free libations, his perceptions took so long a time to germinate that he often burst into laughter at a joke long after it was uttered, and when, perhaps, the topic then on the *tapis* was a death or a funeral.

We have already fully analyzed the oblivion of sleep, the irrationality of the dream of slumber, and its extreme and persistent prototype, insanity. If not, we might perhaps differ with Dr. Holland and Aristotle regarding the period of sleep at which dreams arise, and express our coincidence with Lord Brougham and the author of the "Philosophy of Mystery," that it is the *transition* state. The truth is evidenced by a host of anecdotes related by common dreamers, who were not anxious to prove a position.

We must, however, pass over this chapter, merely alluding to a seeming paradox in page 93: "Some dreams are well remembered, others *not at all*." How do we positively know they occurred, if not remembered? No one but the slumberer can decide this.

And what *is* a dream. This is Bichat's definition:—"Ils ne sont autre chose qu'une portion de la vie animale échappée de l'engourdissement où l'autre portion est plongée." Now no one can deny the plausibility—the truth(?) of this want of balance, or its importance in our study. This *metaphysical* cause, however, leads to no practical result. The vital question seems to be how far the blood, or the nerve, or cerebral *disorganization*, is involved in the states of dreaming and insanity. Dr. Holland refers very acutely to this subject; but, again, we have in many papers so amply discussed it, especially in the comparative importance of blood and nerve, that we must pass by this chapter also, which, according to Dr. Holland's distaste for conjecture, is chiefly bearing on the *difficulties* of the discussion. We may just surmise, that undue *determination* of blood, and of the nervous or sensorial power, have probably about an equal share in many cases. The only allusion we make is to one special exciting cause of insanity, the protraction or excess of reflection; or, as Dr. Holland writes, "the concentration of the consciousness too long continued upon its own functions." The proneness of deep thinkers to ultimate derangement is unhappily not rare; and it is probable that the result is depending on a certain predisposition, which itself may be owing to a peculiar texture of brain or nerve. The firm brain will bear it, and find it but little effort; the softer brain will yield, for the act is *exertion;* always attended, we believe from the beginning, with varied degrees of erethism or excitement.

The pathology of memory ("the reproduction of a perception" of Spurzheim) is of the deepest interest; the faculty, indeed, being constantly exerted by us, even between a question and its immediate(?) answer. There is, in truth there must be, a lapse of time, and, ere we respond, we recollect the question, or we could not answer: of course, therefore, all thought must be memory. Dr. Holland cites some interesting cases of the loss of memory; among them, that of Messala Corvinus, as forgetting his own name; and we might refer to parallel cases of J. W. Von B., &c. &c. Another person lost the memory of words only; events and persons were still remembered. Another patient, on convalescence, constantly substituted one pronoun for another. A third, during his delirium, spoke only in French, a language he had ceased to converse in for thirty years. Priestley, Scott, Porson, and many others of high talent, might also be cited as examples. Many of these cases are paralytics. The pages of our journal, especially in the second volume, and some standard works to which we can refer, abound with these curious records. We cannot agree with Thomas Brown, that voluntary memory or recollection is a mere *suggestion;* if so, it must be from a man to himself: he dictates, when he says, "*let* me recollect." Memory *is* a suggestion; and he says, "I *do* remember." Gall is a little more precise: "Remembrance is the faculty of recollecting that we have perceived impressions; and memory the recollection of the impressions themselves." The *suspension* of memory is one of the most mysterious of mnemonic phenomena; as if, after the intoxication of Lethe, we had corrected it by quaffing copiously of the fountain of Mnemosyne.

Accident, as concussion, or disease, often produces this abeyance, the mind being a mere *tabula rasa,* until, in a moment, when the oppression is removed, thought recurs at once to the point of time when the accident occurred. A rider who has been thrown, will lie in coma even for ten or twelve days, or more, and then directly he comes out of the stupor, will exclaim, "Put the horse in the stable." But we might fill a volume with these anecdotes.

Memory being the essence of all *healthy* thought, its derangement is of course one constant symptom of insanity. But were we to comment on it here, we must of necessity only quote ourselves. Dr. Holland has hinted that "defect of memory may be the primary disease, and insanity its consequence." We have, however, always taken the loss of memory as one prominent sign of disorder of its organ. Returning memory, as the sequela of convalescence, proves this to be the case. And again, the recurrence of certain morbid states of the brain *reproducing* the same distorted image in a patient who had, during the healthy interval, entirely forgotten the illusion, illustrates the intimate dependence of memory on structure.

We cannot attempt to explain that which Dr. Holland waives—the exact mode by which an eidolon is impressed on the brain, or how it can be re-excited. Locke, we remark, asks this question, Whether the temper of the brain makes this difference, in some it being like marble, in others like sand? Haller does not blink the question; and Dr. Hook, one of the first Royal Society Fellows, decides *how many hundred* ideas can be made or retained in one day! It is, however, most probable that *impression* is the proper word, as children retain more than adults. Yet is it also true, that one idea or thought out of so many thousands must displace another, either *confusing* or *dispersing* the rest.

We agree with Dr. Holland, however, on the importance of the study of the varied power of memory in different persons, regarding the system of its *education.* The encouragement of *natural* precocity may often be attended by the same prejudicial effect as the taxing of the memory of children unduly. The constitutional power of the mind, like that of the body, is very varied; and memory, one of the functions(?) of that mind, as well as any physical or clearly organic function, must not be overstrained, or it will of necessity suffer.

*Getting by heart* is to a degree salutary; but if strained, it becomes a sort of parrot learning—very specious perhaps, but it is anything but wisdom. The preceptor, then, should be the physician of the mind, and as watchful as he of the body.

There are, it is true, statements of very extraordinary memories recorded, especially in Sir Alexander Crichton's "Inquiry," and in the "Philosophy of Mystery," &c.; but common powers must not aim at this, or the fable of the bull and the frog may be realized.

Dr. Holland glances at two instances of almost imbecility induced by this over-action; and we should not hazard much if we concluded that varied degrees of ramollissement were the cerebral conditions thus induced. This, we believe, is the gist of Dr. Holland's remarks in page 159 of his work, although he does not quite approve of the word.

Less severe impairment of the memory may, however, be the result of very common causes: a glass of wine may thus restore a failing memory, "so suddenly," as Dr. Holland writes, "as to show that the want of due excitement to the circulation was the cause of the failure."

Thus also in fevers, and all disorders marked by adynamia.

Dr. Holland confesses we have no specific, no mnemosynè for the restoration of fading memory. The only mode must be, to direct remedy to that organic point, of the derangement of which the defect of memory is but a symptom. Restore *completely* the brain, and memory would be sure to return. We refer our readers to the judicious precepts proposed by Dr. Holland as remedial. The effort of recollection will often fail when spontaneous memory succeeds. "A line," Dr. Holland remarks, "laboriously and vainly sought for, will often flash upon the mind when the search has been discontinued." And this seems a curious analogy to the visibility of a small star, when we look *merely at its vicinity*.

Dr. Holland and the late Dr. Wigan (who dedicated his book to him) differ widely on the physiology of brain. Doubleness and duality are very opposite things. Wigan argued that the two minds might antagonize each other—both, in fact, thinking of different subjects *simultaneously*. Dr. Holland's arguments regard succession—a matter of time—the brain thinking on different subjects only at *different* periods: and as it is a *unity*, the two hemispheres are coinciding with each other through the medium of their commissures.

The question is curious, how closely towards duality, division, disease, or absence of these commissures would tend.

Even in alluding to the disturbance of brain and nerve, and even to idiocy, and also to the curious cases of hemiopia, of which Abernethy and others have been subjects, the question of time still reconciles Dr. Holland to mental unity. Perhaps the *immediate succession* of thought may associate fairly the differences of unity, duality, and plurality of mental organization. In page 184 we have very sensible arguments in favour of unity; but whether the one or the other, there must be doubtless *two* states of brain, when a person is irresistibly impelled to that which his better nature abhors. The

> "Video meliora proboque,
> Deteriora sequor,"

and the

> "Contra miglior voler, voler mal pugna"

of Dante, are painful records of the victory of Arimanes over the good spirit. The rapidity of thoughts in succeeding or in galloping over each other will explain all this, and also those curious cases of double consciousness in which we seem to be ourselves and others at one and the same time. Of this interesting question phrenology should be but the alphabet or preface. Phrenology, the doctrine of *mind*, is, however, too much mixed up with craniology, the doctrine of the *skull*, to render its minutiæ yet available for the psychologist. "Viewed as a whole," writes Dr. Holland, "it is a sort of especial contradiction to the *principe de la moindre action*, so generally prevailing throughout all parts of the creation; and it is yet further liable to this peculiar objection, that the limitation of the list of organs is hardly more reasonable than its extent,"&c. The mapping of the skull *quoad* quantity and extent merely, without reference to *quality*, must ever be liable to fallacy, although the unravelling and analysis of brains will doubtless develop much of which we are at present ignorant. Dr. Holland refers with some confidence to the researches of Baillarger in 1845. That phrenologists have jumped precipitately to a conclusion is very clear, and, like many other abstruse or interesting sciences, systems, or processes—mesmerism, vaccination, &c., &c.—phrenology yet suffers from the haste of its sanguine professors in their "over early and peremptory reductions into acts methods," as Lord Bacon writes. In the mass, however, the system is probably true. For the analogy of propensity and instinct, the principle of antagonism of different organs, to the controversion at once of *abstract* phrenology, we refer to Dr. Holland's calm and philosophic reasoning, merely hinting at the fallacy of the mere cranial topography, when we cannot define the direction of convolutions, *in the course of which*, and not across them, we must locate our propensities and faculties.

The line of demarcation between instinct and reason has been by many deemed indefinite; they have been, indeed, identified: but they have really no analogy. Reason is an intellectual act, and progressive; but as man possesses also instinct, so brutes have in a minor degree a reasoning faculty; but instinct is connate and fixed, a custom or habit, as Dr. Holland properly terms it, and no more mental than is the shrinking of the mimosa, or shutting up of the dionæa, perhaps not far removed from the "*affinité des molécules des cristeaux*" of Laplace.

Take instinct then as a sort of inherent vital property, incited by a stimulus, and we may almost reason on it as we would on peristaltic action. We have ever held this to be truth, and therefore coincide in the author's clearly expressed opinions, especially regarding the relative proportions of instinct and reason in man and brute, and also to the inverse perfection of intellect and instinct in the hymenoptera and the hemoptera.

On this point, therefore, we have ever held the hackneyed lines of Pope as a quibble :—

> " And reason raise o'er instinct as you can—
> In this 'tis God directs, in that 'tis man."

If man *does* direct, God has directed man.

There is, therefore, either an inherent law in organization, or a special direction of the creative mind to the organ, for the purpose, which refers an act at once to the Deity, and removes free agency and responsibility. The great and important difference is this : in man reason constantly *controls* instinct; even organic function is fettered by his will. So the powers of resistance are as varied as the temperaments with which they are associated; and we must quote a very luminous passage of our author on this point.

"In considering this curious question of the relation of human instincts to those of lower animals, a valuable distinction may be derived from looking to their respective development in species and individuals. In other animals instincts are chiefly or entirely those of the species, uniform and permanent; with far less of intelligence in any case to modify or control them, and this, at a certain point, disappearing altogether. In man, they have more of individual character, are far less numerous and definite in relation to the physical conditions of life; more various and extensive in regard to his moral nature; yet still subject, as such, to the control of his intellectual powers. It seems the proper destination of reason, as bestowed by his Creator, to acquire mastery over the instinctive conditions of his nature—to cultivate some, to subdue others, to give due proportion and direction to all."

Now, this is one of the most important questions in ethics, inasmuch as this perpetual conflict between the light and shade of human nature involves half the questions of the lunacy commissions and of the criminal court.

It is probable that, regarding erotic and furtive and vindictive propensities, the *vince seipsum* is often as difficult as the *nosce seipsum*. If this, however, were ensured, how less than perfect would man become ! But, alas ! the victory is too often on the dark side; and even if it be not, the climax of the struggle is insanity. Thus, regarding instincts, Dr. Holland writes : "They modify, often control or compel, the whole course of life. In some cases, they are felt as opposed to the reason of the individual, yet dominant over it; in extreme cases they become a sort of madness, by opposition to the reason of the species."

The indications of emotion and the changes of the expression of feeling and passion in different stages of life, involve a curious inquiry, especially as to their being a sort of safety-valve to the system. They —*i. e.*, tears, sighing, even violent efforts, &c.—are doubtless prophylactic in warding off those effects upon organization which might lead to peril or even fatality. They are also a complete study: they are not so explanatory as Le Brun has depicted them, and they are not exactly uniform in every one; still have they a sort of general likeness.

Regarding the earliest instinct, suckling, we may perhaps somewhat differ from the learned author, in deeming it a reflex action. The mother may generally first apply the nipple to the infant's lip, but the lamb certainly rushes at once to the udder of the ewe. Both must be governed by the same law. If the proof of contact always preceded these acts, the reflex theory of Hall would at once untie the Gordian knot. Some instincts are produced, doubtless, by the cerebro-spinal system; but animals who possess it not have instincts highly developed. Even the experiments of Huber and Latreille, to which Dr. Holland refers, still leave the mind unsatisfied. Development or loss of nerves may perhaps in the end decide it. The transmission of habits, mental and bodily, through a succession of races, is another subtile question. It is, of course, possible that certain conditions of nervous or vascular influence may become hereditary, so as to modify the peculiarities of whole communities and races in the course of time (as in the domestication of animals), so that the constitution of mind may "become hereditary by propagation." It is, indeed, an acquired or second nature. In proof of this, Dr. Holland refers to hereditary monstrosity, and inclines to the belief of some structural constitution or quality. In the case of brute animals, we cannot believe in the working of mental impression or propagation, notwithstanding the incident of Laban's sheep: but in the cases of monstrosity it may be that the brooding over an unnatural state of one member of a family may, in the pregnant mother, induce a sort of uterine erethism in several successive generations; while the potent influence of imitation in the transmission of peculiarities and eccentricities is a truth constantly before us.

The nervous system—what a deep and intricate study! what a mine of physiological riches yet unexplored, after the myriad of disquisitions, from Aristotle even to our own day! Its influence is universal—the source of our perceptions, our consciousness, our reflections, pleasure, pain, and sympathies. Dr. Holland shrewdly foresees the difficulty of ascertaining the nature or mode of this influence. The opinion that sensation and volition simply depend on centripetal and centrifugal nervous action; the varied phenomena, as secretion, volition, reflex action, &c., brought out in different tissues by the same apparent nervous causes; and the functions of organic life,—are referred to as proofs of this. But still the learned author looks the question fairly in the face, if he has not decided on the nature or *matériel* of nervous influence, whether a subtile fluid, an electric aura, or "some superior and independent principle, of which, however designated, the brain is the immediate source or seat." But the recognition even of this principle, as well as the new sense of the Germans, *selbstgefühl*, and even the grand philosopher's stone of physiology—the vital principle—would, probably, even if demonstrated, leave neurology somewhere about where we left it. The theologian and the physiologist being still at issue, the theory of vitality—the breath of life—the living soul—the quickening spirit—*νους*—*θυμοσ*—*ψυχη*, and other Greek terms, will still all be bandied about, like battledore and shuttlecock, between the combatants, even as the vital principle was in the arena of the College of Surgeons, between two learned professors, in days not long agone. It has ever seemed to us that the analysis of spontaneous generation, or parthenogenesis, of the lower vitalities, indicates the simplicity of a principle which becomes complex, and indeed confused, in proportion as organization, of which it is the moving power, becomes more intricate or combined. We know not which it is that in old age wears out, according to its law, the principle or the organization, any more than we know whether nervous power influences the germ, and expands with it, or if it be *generated* by the nerve in the progress of its growth. We do know, however, that, as the nervous system is multiplied or expanded, the phenomena are themselves extended or refined. The minute anatomy of Ehrenberg, the ingenious though somewhat fanciful notions of Alison, and the host of modern English physiologists even now prosecuting their labours, added to the deductions of deep thinkers like our author, will continue to throw fresh light on the corridors and chambers of science, even if they do not illumine the very *sanctum sanctorum* of the temple.

How far the hemispherical ganglion and the tubular neurine are concerned in the generation and transmission of psychal phenomena, is of the extremest importance to our subject. But perhaps the most important matter of all is, the intimate relation which anatomy discovers between nerve and blood-vessel, which, indeed, involves all the mysteries and discrepancies of psychology. The lens can never ultimately divide that which is infinitely divisible; but as this applies as well to one as to the other, we may still discover very much as to their dominant or prevailing influence in psychology, and perhaps thus ascertain many truths regarding the *primal impingement* of malaria.

Dr. Holland, perhaps wisely, leaves this deep question with a passing glance at its importance, and proceeds to a general view of the three grand divisions of the nervous system—viz., the cerebrum and cerebellum, .the cerebro-spinal axis, and the sympathetic. The cerebro-spinal, the tract between brain and body, will, we conjecture, be the great Californian field whence the gold dust of psycho-physiology will be, for the present, gathered; especially regarding remote sympathies, one curious instance of which was noticed by Bernard, of diabetes induced by injury to this part.

Dr. Holland believes that "memory and association are more closely related to the cerebral hemispheres than any other attributes of mind." The unravelling of the cerebral convolutions, he thinks, "detaches mind itself from all material organization." It resembles, we suppose, a peep behind the curtain, which dispels the illusion of the scenic representations. The contradiction, by *post-mortem* evidence, of the received hypothesis, especially regarding the function of the cerebellum as an organ of sexual impulse, or a regulator of muscular movements, imperatively requires strict investigation. This, and the physiology of the sympathetic system, Dr. Holland terms a *terra incognita.* We confess we have little hope of soon rendering the circulation of nervous fluid as plain as that of the blood. In the mean time, innervation exhibits so many analogies to electric forces, that our minutest attention should be paid to the resemblance. Dr. Holland almost describes this analogy when he writes of "a power originating within the system, and transmitted, *progressively,* along the course of the nerves, to fulfil its functions in the several parts of the body to which they conduct."

We have, like Dr. Holland, seen several cases in which there is a general "deficient evolution of nervous power." He refers to two or three cases of his own; and we may allude, also, to the case of Cowper. All these are, however, but extremes of what daily occurs. In the senses also, in prejudices or antipathies; in the collapse consequent to surgical operation, or shock; in the slumber between the pangs of lingering labour; and in the criminal on the night before his execution, we see this enervation, arising from the exhaustion either of excess of sensibility or of suffering.

The curious effects of chloroform have seemed to us illustrations of this rapid exhaustion, rather than of direct anodyne influence. In most cases (and we have seen one in a boy on the day we are writing this), the first influence is excitement, somewhat like that from nitrous oxide inhalation; it may even amount to convulsive action, and then speedily comes on the collapse both of sensory and motive power; indeed, a state of trance.

This bears Dr. Holland out in his notion of *quantity* in reference to innervation, not only in defect but also in excess, as in the irresistible and almost supernatural and disproportionate spasmodic actions, in which it is, indeed, often perilous suddenly to close this safety-valve of the system.

This subject of innervation strikes, also, deeply at the root of insanity; and we are pleased to observe the coincidence of the author with our recorded opinions. The allusions to excess, defect, intensity, quality, and time, evince his close and prudent reasoning.

Then comes the popular question—which again we have prejudged, especially in our review of Catherine Crowe.

On the *intercommunication* of nervous influence, some will persist in saying, if not in believing, consist the truths of mesmerism and electro-biology. We think (and Dr. Holland evidently thinks so too) that, notwithstanding the experiments of Reymond in inducing deviations of the needle by muscular action, there is no necessity for this *projectile* faculty of mind or nervous power, or of the blue fluid of Dupotet, for the explanation of the phenomena: indeed, one *self-made* experiment in electro-biology would, we think, settle the question.

It will be seen how much of Dr. Holland's volume is taken up in referring to the difficulties of his subject. So much simplicity and beauty, however, are displayed in his reasoning, that we almost regret the guarded opinions and reluctance to form conclusions which ever mark the writings of a true philosopher. So, while the book raises, throughout, our admiration of its purity, and indeed of its humility, it makes us the more confess, with Socrates, and (may we say?) Dr. Holland, that all our knowledge consists in our knowing that we know nothing.

